# Supplementary figures and images for: The transcription and export complex THO/TREX contributes to transcription termination in plants
Source: PLoS Genet. 2020 Apr 13;16(4):e1008732. doi: 10.1371/journal.pgen.1008732 (PMC7179932; doi:10.1371/journal.pgen.1008732)

**A**

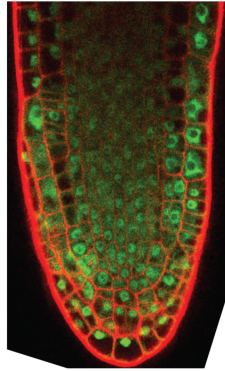

pTEX1::gTEX1::GFP

**B**

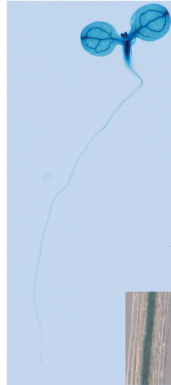

Seedling

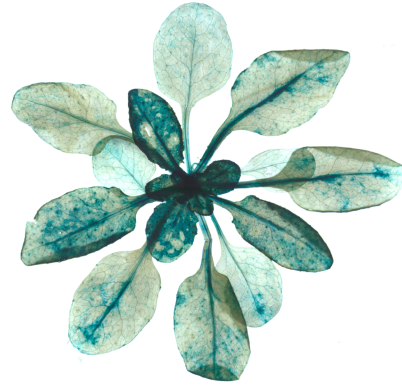

Rosettes

pTEX1::GUS

**C**

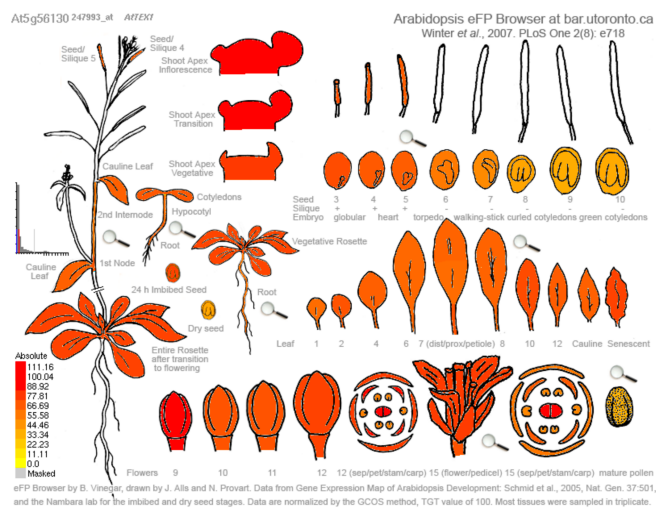

Supplement: S1 Fig — (A) TEX1 localization in root tips of 5-day-old seedlings. Expression of TEX1::GFP fusion is under the control of the endogenous TEX1 promoter. (B) GUS expression from the TEX1 promoter in roots and cotyledons of 5-day-old seedlings (left) as well as rosette leaves of 4-week-old plants (right). Plants were transformed with a TEX1 promoter:GUS construct. Inset (lower right) shows expression in a section of mature root. (C) Expression profile of the TEX1 gene in Arabidopsis as visualized with the eFP Browser 2.0 (https://bar.utoronto.ca/efp2/). (PDF) [file pgen.1008732.s001.pdf]

*pho1-7tex1-4* vs *pho1-7* in pot

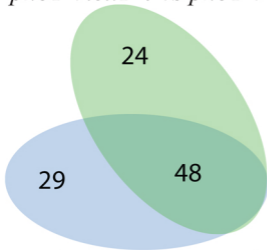

*tex1-4* vs Col0 in petris

Supplement: S4 Fig — RNA extracted from roots of pho1-7 tex1-4 and pho1-7 plants grown in pots for 4 weeks (green) or tex1-4 and Col-0 plants grown in petri dishes for 7 days (blue) were used. (PDF) [file pgen.1008732.s004.pdf]

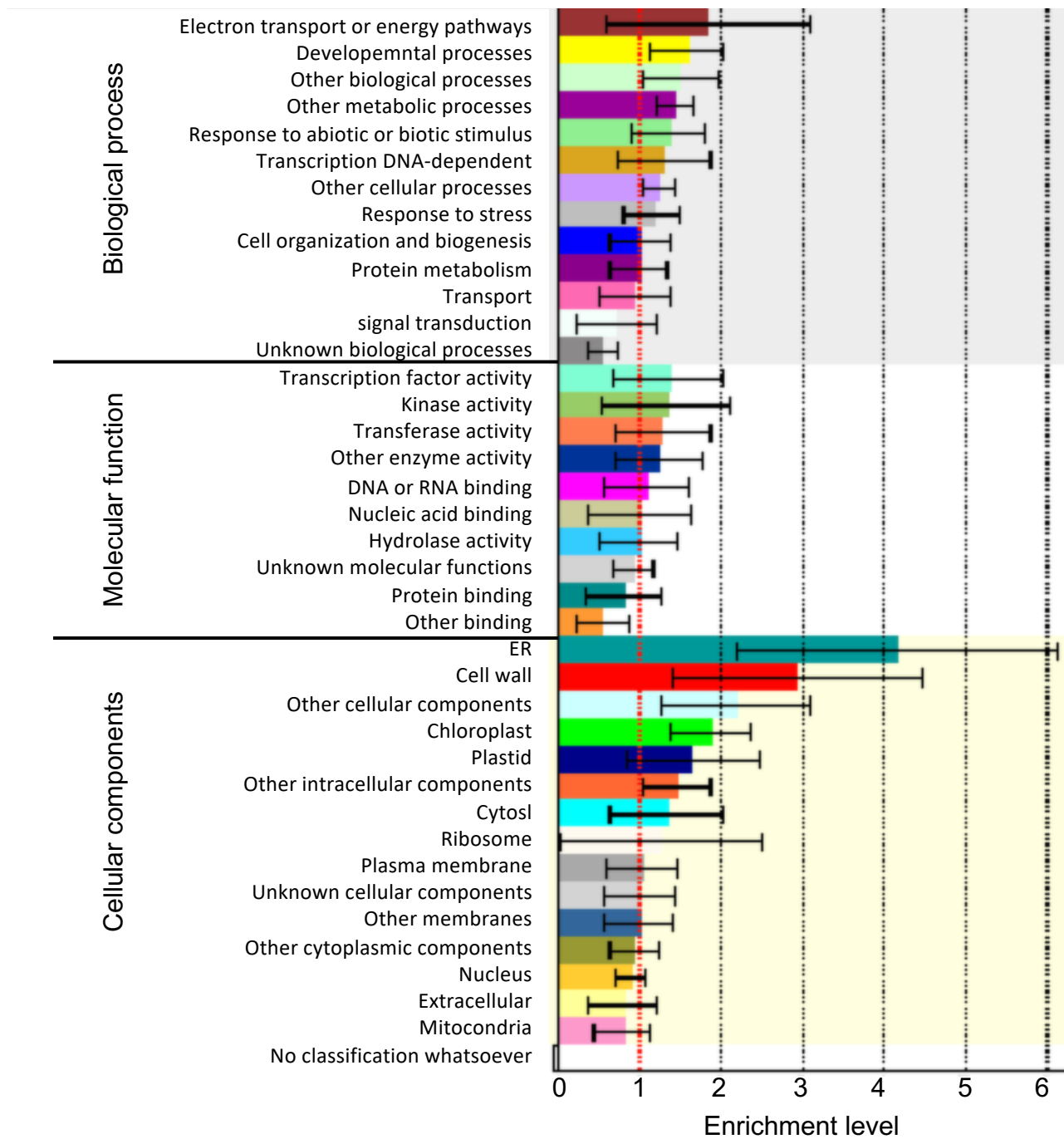

Supplement: S5 Fig — The histograms show the fold enrichment of a given gene ontology term. (PDF) [file pgen.1008732.s005.pdf]
