# Supplementary material for: The transcription and export complex THO/TREX contributes to transcription termination in plants
Source: PLoS Genet. 2020 Apr 13;16(4):e1008732. doi: 10.1371/journal.pgen.1008732 (PMC7179932; doi:10.1371/journal.pgen.1008732)
Supplement: S2 Fig — (A) Relative expression of phosphate starvation-induced genes in the shoots of 4-week-old plants. (B) Lipid quantification in the shoots of 4-week-old plants. DGDG, digalactosyldiacylglycerol; PG, phosphatidylglycerol; PE, phosphatidylethalonamine. Data in A and B are means of three samples from plants grown in independent pots and three technical replicates. Error bars represent standard deviation. Values marked with lowercase letters are statistically significantly different from those for other groups marked with different letters (P < 0.05, ANOVA with the Tukey-Kramer HSD test). (PDF) [file pgen.1008732.s002.pdf]

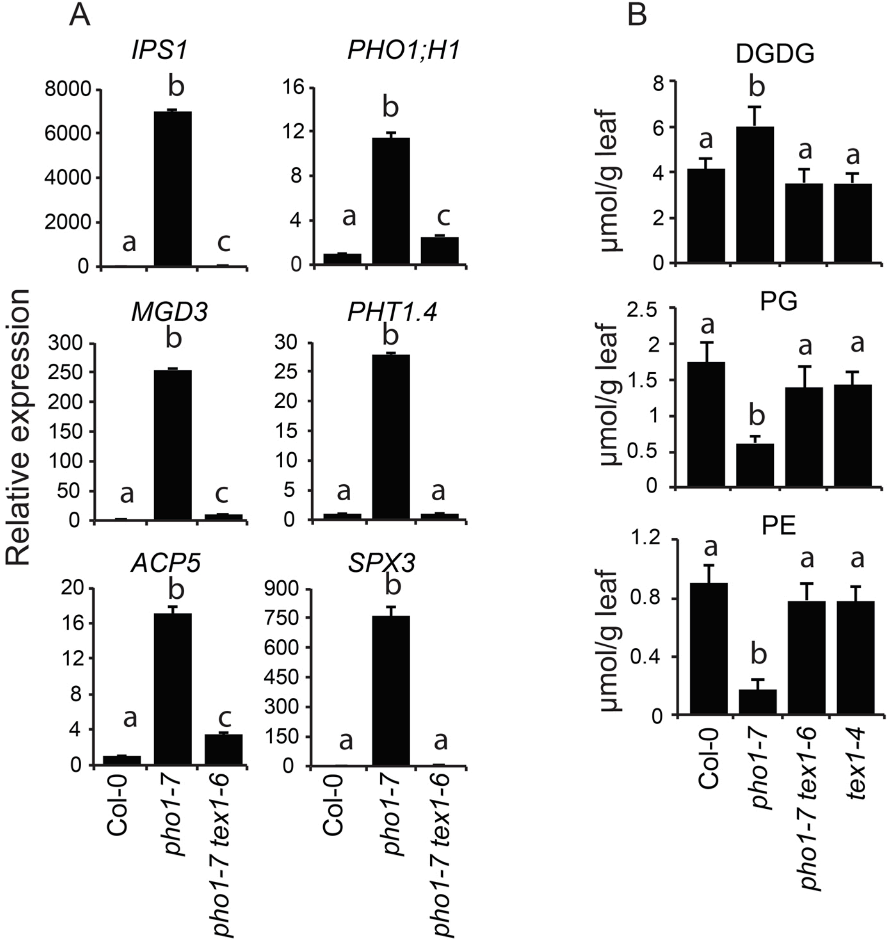
A
B
IPS1
PHO1;H1
MGD3
PHT1.4
ACP5
SPX3
Relative expression
DGDG
PG
PE
µmol/g leaf
Col-0
pho1-7
pho1-7 tex1-6
tex1-4
