# Supplementary material for: The transcription and export complex THO/TREX contributes to transcription termination in plants
Source: PLoS Genet. 2020 Apr 13;16(4):e1008732. doi: 10.1371/journal.pgen.1008732 (PMC7179932; doi:10.1371/journal.pgen.1008732)

Illumina sequencing

A.

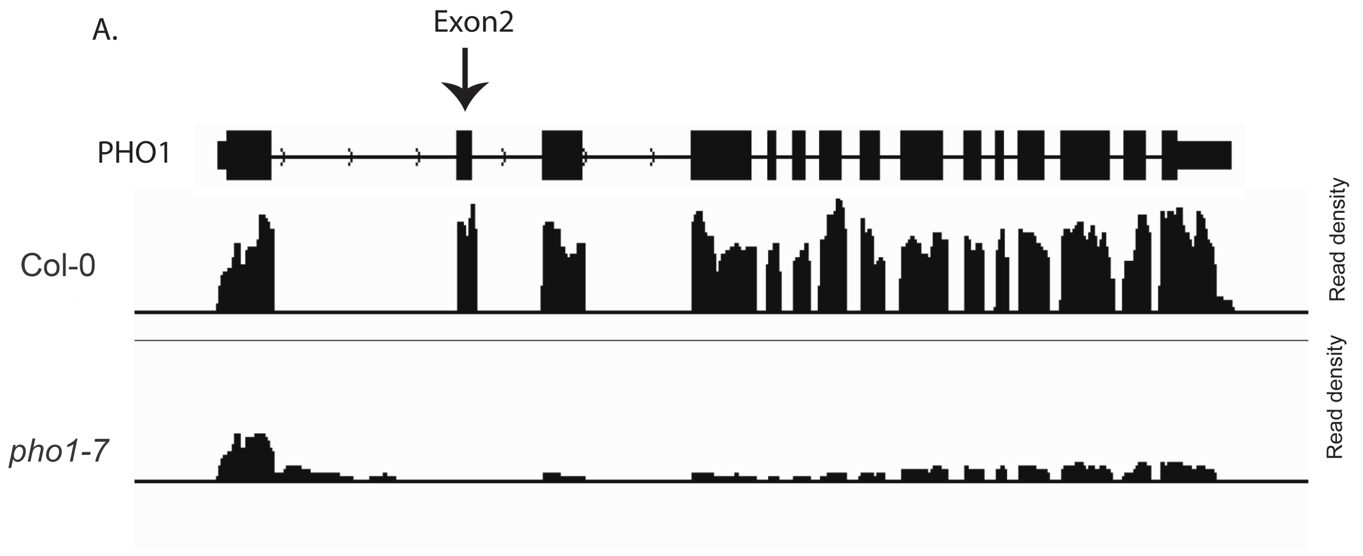

Pac-Bio sequencing

B.

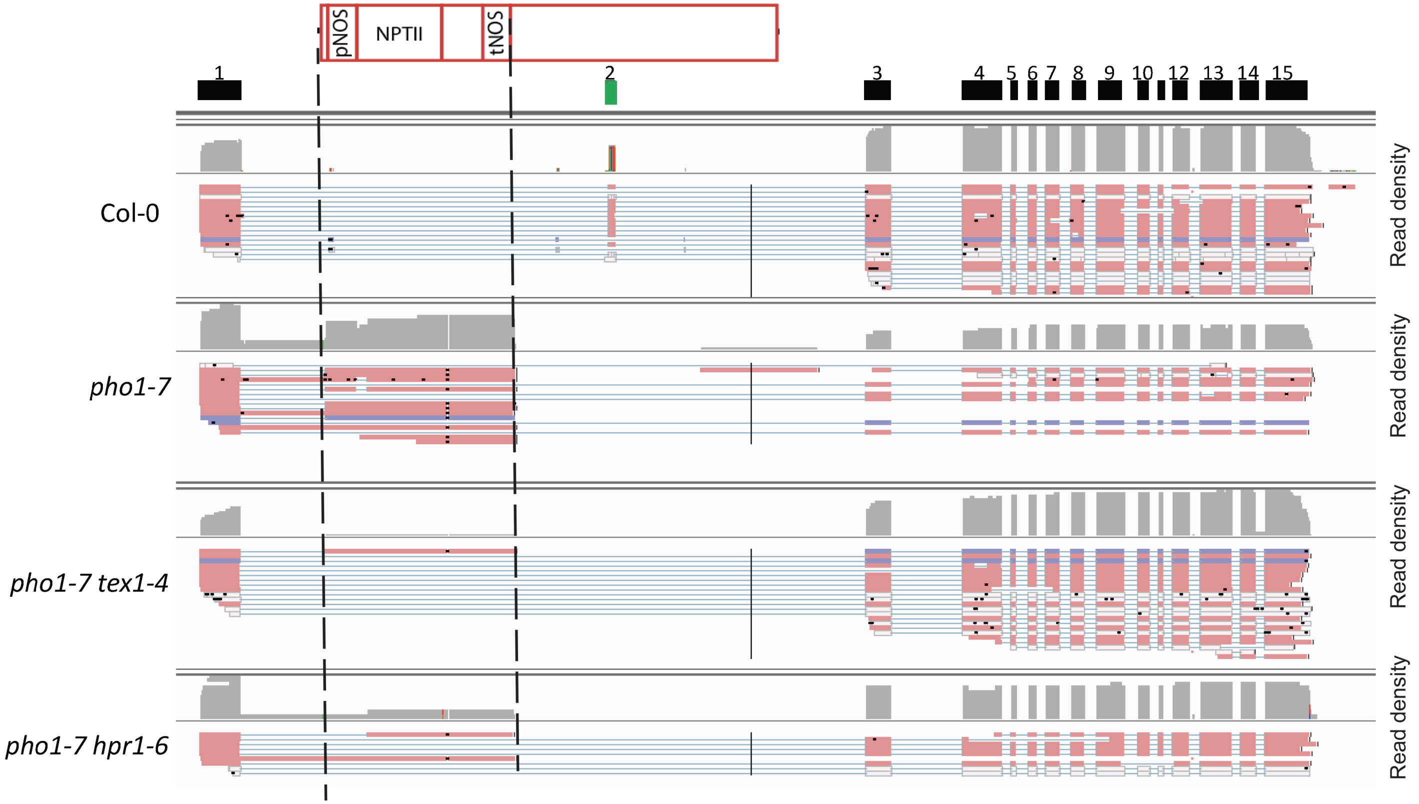

Supplement: S3 Fig — (A) Illumina RNA sequencing reads density graph mapped against the wild-type PHO1 locus. Note that exon 2 of PHO1 is missing in the pho1-7 mutant because of T-DNA insertion while it is present in Col-0. (B) Pac-Bio read density graph showing full length mRNA structure at the PHO1 locus. The top red box shows the location of the T-DNA in pho1-7. pNOS, NOS promoter; NPTII, neomycin phosphotransferase gene; tNOS, NOS terminator. The black boxes below shows the positions of the 15 PHO1 exons, except for exon 2 which is shown in green. Exon 2 is present in Col-0 but deleted in the pho1-7, pho1-7 tex1-4 and pho1-7 hpr1-6 mutants as a result of T-DNA insertion. For each genotype, the sequence of independent cDNAs are shown by individual lines (red and blue lines) with the grey areas representing the sequence density in each region. (PDF) [file pgen.1008732.s003.pdf]
